# *e*PIANNO: ePIgenomics ANNOtation tool

**DOI:** 10.1371/journal.pone.0148321

**Published:** 2016-02-09

**Authors:** Chia-Hsin Liu, Bing-Ching Ho, Chun-Ling Chen, Ya-Hsuan Chang, Yi-Chiung Hsu, Yu-Cheng Li, Shin-Sheng Yuan, Yi-Huan Huang, Chi-Sheng Chang, Ker-Chau Li, Hsuan-Yu Chen

**Affiliations:** 1 Institute of Statistical Science, Academia Sinica, Nangang, Taipei, Taiwan; 2 Bioinformatics Program, Taiwan International Graduate Program, Academia Sinica, Nangang, Taipei, Taiwan; 3 Institute of Biomedical Informatics, National Yang-Ming University, Taipei, Taiwan; 4 Department of Clinical Laboratory Sciences and Medical Biotechnology, College of Medicine, National Taiwan University, Taipei, Taiwan; 5 NTU Center for Genomic Medicine, National Taiwan University College of Medicine, Taipei, Taiwan; Osaka University, JAPAN

## Abstract

Recently, with the development of next generation sequencing (NGS), the combination of chromatin immunoprecipitation (ChIP) and NGS, namely ChIP-seq, has become a powerful technique to capture potential genomic binding sites of regulatory factors, histone modifications and chromatin accessible regions. For most researchers, additional information including genomic variations on the TF binding site, allele frequency of variation between different populations, variation associated disease, and other neighbour TF binding sites are essential to generate a proper hypothesis or a meaningful conclusion. Many ChIP-seq datasets had been deposited on the public domain to help researchers make new discoveries. However, researches are often intimidated by the complexity of data structure and largeness of data volume. Such information would be more useful if they could be combined or downloaded with ChIP-seq data. To meet such demands, we built a webtool: ePIgenomic ANNOtation tool (*e*PIANNO, http://epianno.stat.sinica.edu.tw/index.html). *e*PIANNO is a web server that combines SNP information of populations (1000 Genomes Project) and gene-disease association information of GWAS (NHGRI) with ChIP-seq (hmChIP, ENCODE, and ROADMAP epigenomics) data. *e*PIANNO has a user-friendly website interface allowing researchers to explore, navigate, and extract data quickly. We use two examples to demonstrate how users could use functions of *e*PIANNO webserver to explore useful information about TF related genomic variants. Users could use our query functions to search target regions, transcription factors, or annotations. *e*PIANNO may help users to generate hypothesis or explore potential biological functions for their studies.

## Introduction

Transcription factors (TFs) and chromatin regulators (CRs) play key roles in eukaryotic gene transcriptional processes[1, 2]. Mapping the genomic locations of these factors is crucial to understand the mechanisms of transcriptional and epigenetic regulation. Recently, with the development of next generation sequencing (NGS), the combination of chromatin immunoprecipitation (ChIP) and NGS, namely ChIP-seq, has become a powerful technique to capture potential genomic binding sites of regulatory factors, histone modifications and chromatin accessible regions[3, 4]. Many ChIP-seq datasets had been deposited on the public domain to help researchers make new discoveries. However, researches are often intimidated by the complexity of data structure and largeness of data volume.

Currently, many ChIP-seq web servers and databases have been developed to provide solutions for diverse biological issues. For examples, CR Cistrome is a ChIP-seq database for chromatin regulators and histone modification linkages in human and mouse [5] and ChIPBase is a database for decoding the transcriptional regulation of long non-coding RNA and microRNA genes from ChIP-Seq data [6]. NCBI Gene Expression Omnibus (GEO) [7] and Sequence Read Archive (SRA) [8] are the most popular depositories of public domain but they do not provide function for interactively exploring ChIP-seq data. ChIP-X database [9] and Factorbook.org[10] collect targeted genes of TFs from published ChIP-seq data but tools for retrieving data across samples are not supported currently. Several resources provide annotated metadata for ChIP-Seq or DNase-Seq studies such as nuclear receptor CistromeFinder, Cistrome, NCBI Epigenomics, hmChIP, SwissRegulon, and CistromeMap [11–17]. Most of them only support the gene symbol querying method and only reveal background information of samples or studies. Although UCSC browser supported multiple types of query and results are shown in an interactive graph, it currently does not provide any batch query function. In addition, previous studies did provide valuable results and information of ChIP-seq data for researchers [18–20]. However, researchers without computational abilities cannot explore associations between their interesting TFs, binding sites, or genomic variations by themselves.

In this work, we considered two important databases that have been frequently used in the genetic research. The first one was the 1000 Genomes Project which provided an overview of human genomic variation, mainly single-nucleotide polymorphisms (SNPs) and small InDels [21, 22]. A total of 1,092 individuals from 14 populations in four continents of the world were enrolled to this project and phase 1 data was released in 2012. The 1000 Genomes Project reveals the alternative allele frequency (AF) distribution of population. A genomic variant with high AF in human general population is considered as a common variant while one with skewed distribution cross different populations would be inferred as a population specific variant. The second database we considered was Genome-Wide Association Studies (GWAS) Catalog of National Human Genome Research Institute (NHGRI) [23] which mainly provided the SNP-trait association data. A total of 11,912 SNPs and 1,060 diseases from 1,751 published GWAS were enrolled.

For most researchers, additional information including genomic variations on the TF binding site, allele frequency of variation between different populations, variation associated disease, and other neighbour TF binding sites are essential to generate a proper hypothesis or a meaningful conclusion. Such information would be more useful if they could be combined or downloaded with ChIP-seq data. To meet such demands, we build an integrative ChIP-seq webserver: *e*PIANNO (http://epianno.stat.sinica.edu.tw/index.html). *e*PIANNO contained over 1000 samples from over 3000 ChIP-seq experiments of human. *e*PIANNO a had a user-friendly website interface allowing researchers to explore, navigate, and extract data quickly. It provided two query functions including official gene symbol and local region, respectively. User could query and extract peak data across samples and experiments in small regions they interest. Results of each query were annotated by 1000 Genomes Project phase 1 release, GWAS Catalog of NHGRI[23], and annotation of ENCODE[24]. *e*PIANNO may help researches to explore potential biological functions of the TFs in which they were interested, thereby helping them generate hypothesis for future studies.

## Materials and Methods

*e*PIANNO was constructed by combining the ChIP-seq data part and the annotation part. In the ChIP-seq data part, data came from three sources. The first one was hmChIP database containing 392 ChIP-seq experiments of human including 129 chromatin modification (CM) and 263 transcription factor binding site (TFBS) experiments (http://jilab.biostat.jhsph.edu/database/cgi-bin/download.pl) [13]. The second was ENCODE database containing 1654 ChIP-seq experiments of human including 489 CM and 1165 TFBS experiments (http://genome.ucsc.edu/ENCODE/downloads.html). The third one was Roadmap Epigenomics Project containing 1,069 CM experiments (http://genboree.org/EdaccData/Release-9/). The peak calling step had been conducted by hmChIP, ENCODE, and ROADMAP Epigenomics, respectively. We only curate the peak files (chromosome, start, and end positions) of ChIP-seq datasets from three projects and provide original sources. In hmChip, datasets were collected from GEO database in BED file format contained chromosome, start, and end positions. However, they stop updating in 2011 and the genome mapping version was hg18. As a consequence, we remapped the BED file provided from hmChip to hg19. In ENCODE, four peak callers, SPP, GEM, PeakSeq, and MACS, were integrated in their uniform peak calling pipeline to generate final peak file. In ROADMAP epigenomics, Pash 3.0 read mapper was used to make reads mapping first. For the histone ChIP-seq data, the MACSv2.0.10 peak caller was used to identify peak region (https://github.com/taoliu/MACS/). For DNase-seq data, the Hotspot algorithm was used to identify fixed-size (150bp) DNase hypersensitive sites and MACSv2.0.10 was used to call peaks using the same settings as the histone mark. Fragment lengths for each dataset were pre-estimated using strand cross-correlation analysis and the SPP peak caller package (https://code.google.com/p/phantompeakqualtools/) and these fragment length estimates were explicitly used as parameters in the MACS2 program (—shift-size = fragment_length/2). Totally, ePIANNO contained potential 253,000,853 peaks in various cell types or experiments. Genomic variant data from 1000 Genomes Project [21], GWAS Catalog of NHGRI[23], and gene annotation of ENCODE[24] were included in the annotation part (Table 1).

**Table 1:** Databases and data size in the system.

Database Name	Feature	Experiments	Data size
hmChIP	CM^ⱡ^	129	3,494,199 peaks
	TF^ⱡ^	263	6,235,702 peaks
ENCODE	CM^ⱡ^	489	33,690,989 peaks
	TF^ⱡ^	1165	33,809,568 peaks
Roadmap Epigenomics	CM^ⱡ^	1069	175,770,395 peaks
NHGRI	Number of disease		663
	Number of unique SNP		7,883
1000 Genomes Project	Number of individual		1,092
	Number of SNP		36,820,992
ENCODE annotation	Number of gene		40,686

Databases and data size of the *e*PIANNO. For ChIP-seq data part, peak data from hmChIP, ENCODE, and Roadmap Epigenomics databases were recruited in the system. Totally, the database contained over 253 million peak data in various cell types or experiments. For annotation part, there were 36,820,992 genomic variant data from 1000 Genomes Project, 7,883 unique SNPs and 663 diseases from GWAS Catalog of NHGRI, 40,868 annotations of gene from ENCODE annotation, respectively.

^ⱡ^ CM: chromatin modification; TF: transcription factor

## Results

### Most disease-associated SNPs locate in the protein-binding region

**Fig 1:**
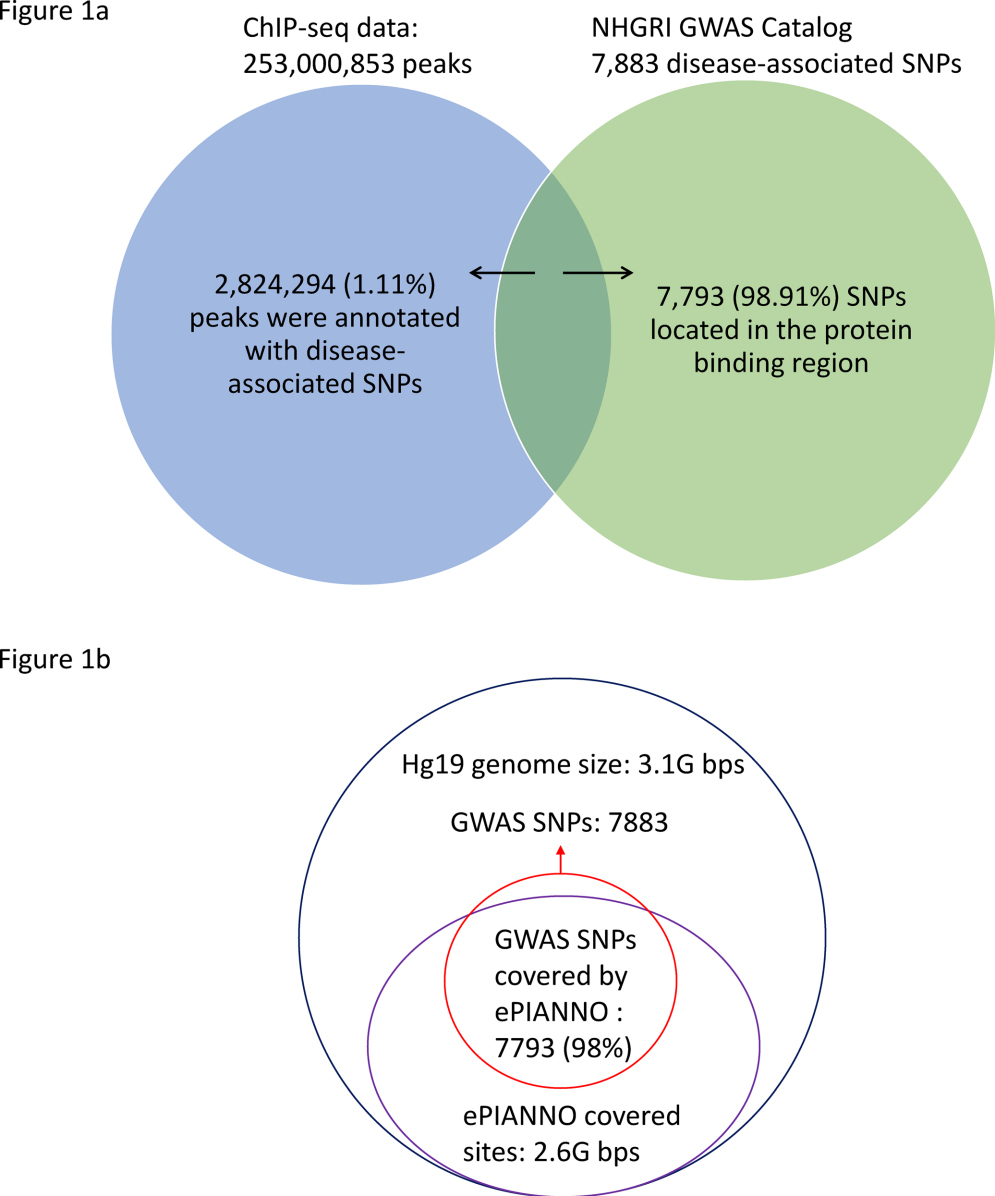
Disease-associated SNPs in *e*PIANNO. (a) A total of 7,793 (98.91%) out of 7,883 SNPs were covered by peaks recruited in *e*PIANNO. (b) Totally 2,668,537,612 bps (86.65%) were covered by Chip-seq experiments based on hg19 genome size (3,079,843,747 bps). A hypergeometric test showed that disease-associated SNPs were enriched in the ChIP-seq covering regions (*P* < 2E-16).

Among all potential DNA binding sites in the *e*PIANNO, 2,824,294 (1.11%) potential DNA binding sites had disease-associated SNPs annotated by the GWAS Catalog of NHGRI. Among all disease-associated SNPs annotated by the GWAS Catalog of NHGRI, 7,797 (98.91%) SNPs were found in the potential protein-DNA binding regions (Fig 1A). This high percentage of disease-associated SNPs may imply that the protein-DNA binding event is crucial to reveal the associations between genomic variants and diseases.

We calculate total sites and disease-associated sites covered by all ChIP-seq experiments collected in ePIANNO. Totally 2,668,537,612 bps (86.65%) were covered by Chip-seq experiments based on hg19 genome size (3,079,843,747 bps). This result showed that although the overall lengths of all peaks in ePIANNO was 206 billion bps, most of them (98%) were overlapped with each other. A hypergeometric test showed that disease-associated SNPs were enriched in the ChIP-seq covering regions (*P* < 2x10^-16^, Fig 1B). This result is smiliar with a previous analysis study done by Bryzgalov et al. They describe an approach aggregating the whole set of ENCODE ChIP-seq data in order to search for regulatory SNPs and concluded that SNPs located in the protein-DNA region are more likely to influence transcription regulation[19].

### Utility and web interface: query and retrieval

Currently, *e*PIANNO provided four functions: searches of transcription factor, target gene, correspondent binding region, and gene annotations. User could search the database by official gene symbol (ex. *EGFR*) or by coordinate (ex. chr7:10000~10000000). When users search specific peaks or genes, both forward and reverse streams of genomic regions will be reported and results will be shown on the browser. *e*PIANNO also provided one-click download function for user to retrieve results in EXCEL CSV format.

### Search transcription factors

**Fig 2:**
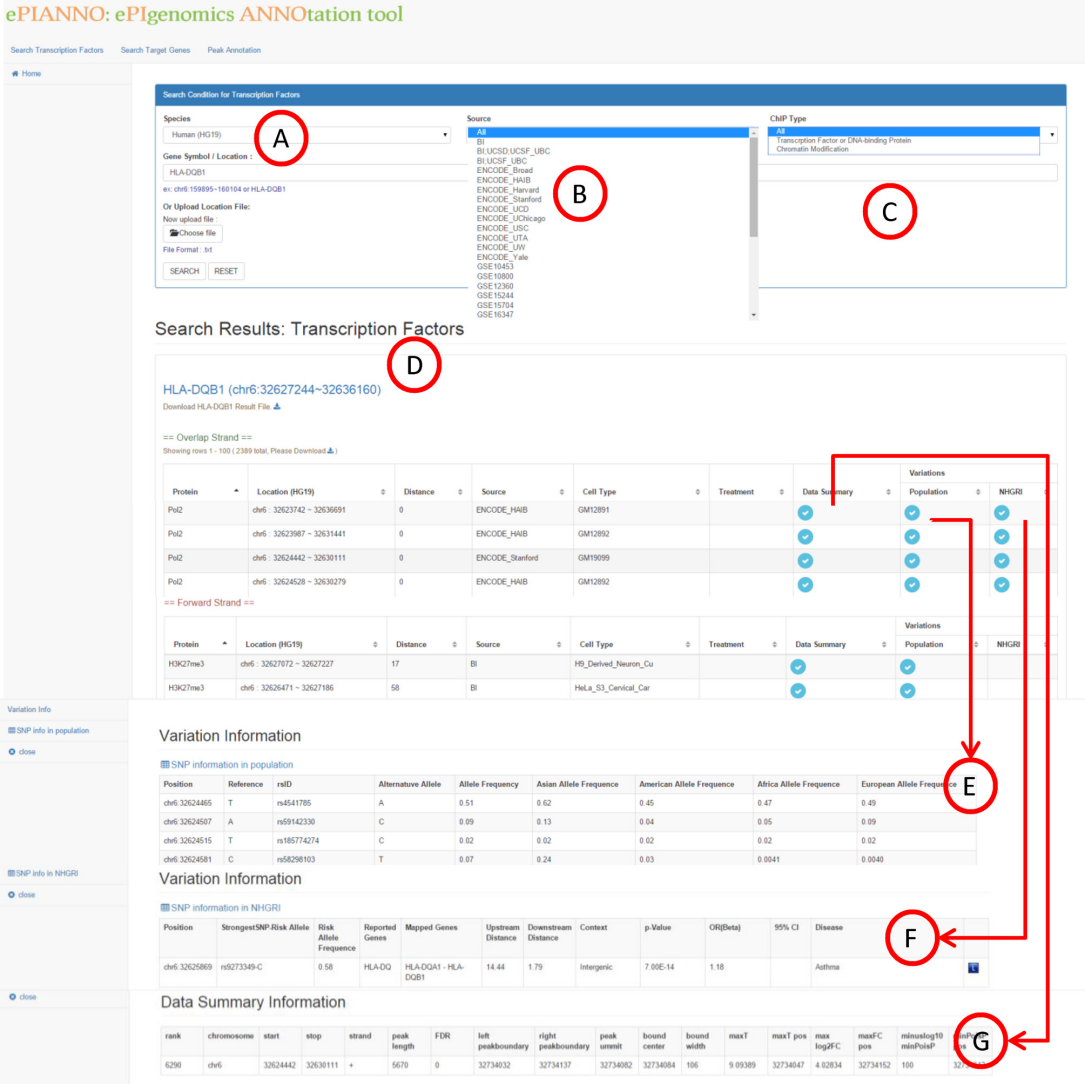
Search transcription factors. If user wants to know potential TFs that regulate gene or genomic region of interest, they could specify gene name, ex., or genomic region, ex. “chr6:159895~160104” or “HLA-DQB1” in the query box (A). User could choose specific source of experiments (B) or ChIP type (C). Results including transcription factor/chromatin modification names, location, distance to gene, data source, and sample name will be shown on the same web page (D). User could expand advanced results by clicking buttons “Variation” for SNP information in populations (E), disease related variants(F), and “Data Summary” for peak data of the ChIP-seq experiment (G).

The first function of *e*PIANNO is the search of TFs. If user wants to know potential TFs that regulate a gene of interest, *e*PIANNO will search TFs whose DNA-binding locations were around the gene of interest based on peak information of ChIP-seq experiments. The first step is to input the specific gene in the query box (Fig 2A). User could choose specific source of experiments (Fig 2B) or a ChIP type (Fig 2C). Basic results including transcription factor/chromatin modification names, location, distance to gene, data source, and sample name will be shown on the same web page (Fig 2D). By clicking the correspondent buttons, a user could expand the detail of results including SNP information in population (Fig 2E), disease related variants (Fig 2F), and peak data of the ChIP-seq experiment (Fig 2G) in peak region. “SNP information in populations” contains information of SNP coordinate, allele call, and minor allele frequency in populations of the four continents. “SNP information in NHGRI” contains SNP-trait association data with correspondent PubMed link from GWAS Catalog of NHGRI. Finally, peak summary shows data of ChIP-seq experiment including peak length, start and end site of peak, and other information in original data source. For example, if a user wants to explore what TFs may regulate *HLA-DQB1*, click “Search Transcription Factors” button at the top-left of the web page and put “*HLA-DQB1*” in the query box. *e*PIANNO will show all TFs binding locations in up or downstream of *HLA-DQB1*. Advance results such as peak data summary and variations could be expanded by clicking the button. In addition, if a user only focuses on specific genomic region instead of gene, *e*PIANNO also shows binding locations and correspondent TFs in the given genomic region. For example, a user can specify genomic region, such as “chr6:159895~160104”, or multiple regions separated by semi-colon or another line, such as “chr6:159895~160104; chr6:182232~210148”, in the query box. *e*PIANNO will show information of TFs, variations, and peak data similar with results mentioned above.

### Search target genes

**Fig 3:**
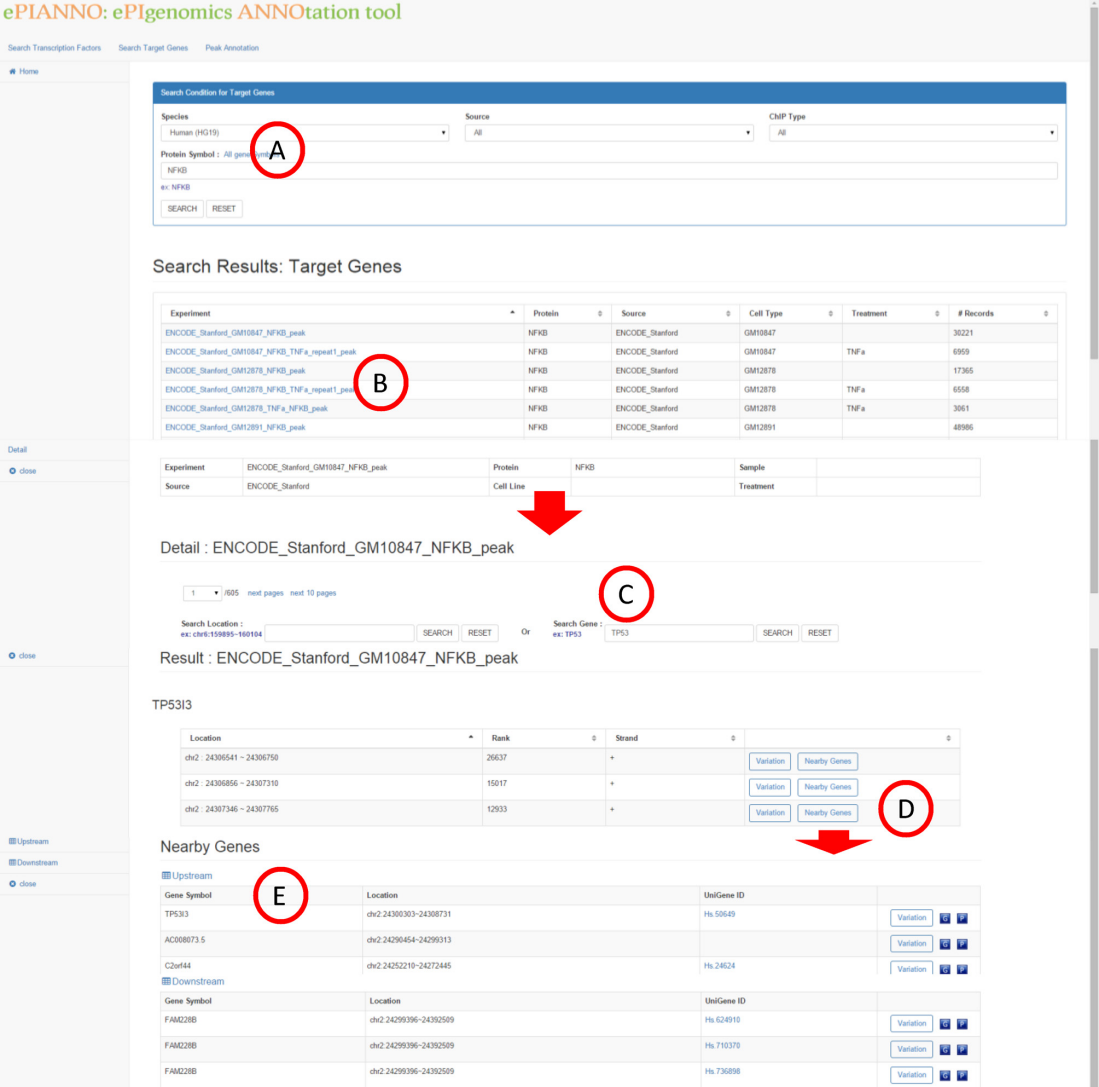
Search target genes. Given a TF name of interest in the query box (A), *e*PIANNO shows DNA-binding locations of this TF based on peak data from ChIP-seq experiments (B). Additional information of DNA-binding locations (C) and detail information of the location (D) will be shown in a new window. Two buttons could be further clicked, “Variation” and “Nearby Genes”. “Variation” shows all SNPs in the binding location and their association with diseases. “Nearby Genes” shows genes located upstream or downstream of the binding location (E).

The second function of *e*PIANNO is to search target genes of a TF. Given a TF name of interest (Fig 3A), *e*PIANNO shows DNA-binding locations of this TF based on peak data from ChIP-seq experiments (Fig 3B). A user could find additional information of DNA-binding locations (Fig 3C) through clickable links and the detail information of the location (Fig 3D) will be shown in a new page. However, the candidate binding regions may be over thousands and further filtering is needed. In this new page, user could further narrow down the candidate list by specifying the target region or gene. Two buttons could be further clicked, “Variation” and “Nearby Genes”. “Variation” shows information of all SNPs in the binding location including their association with diseases. “Nearby Genes” shows genes located nearby upstream or downstream of the binding location (Fig 3E). These genes may be potentially regulated by the queried TF (Fig 3A).

For example, inputting the TF name “*NFKB*” in the query box, ePIANNO will show all ChIP-seq experiments of *NFKB*. After clicking one of experiment link, candidate binding regions of *NFKB* will be shown in a new page. In this new page, user could further specify a gene or a region of interest to explore whether it was bind by *NFKB* or not. Advanced results of the DNA-binding location showed SNP-disease association information, SNP information of populations, and genes located nearby upstream or downstream of the DNA-binding location. These genes nearby the DNA-binding location of *NFKB* may be potentially regulated by *NFKB*. Furthermore, SNP-disease association information and SNP information of populations of these genes are also shown.

### Peak annotations

**Fig 4:**
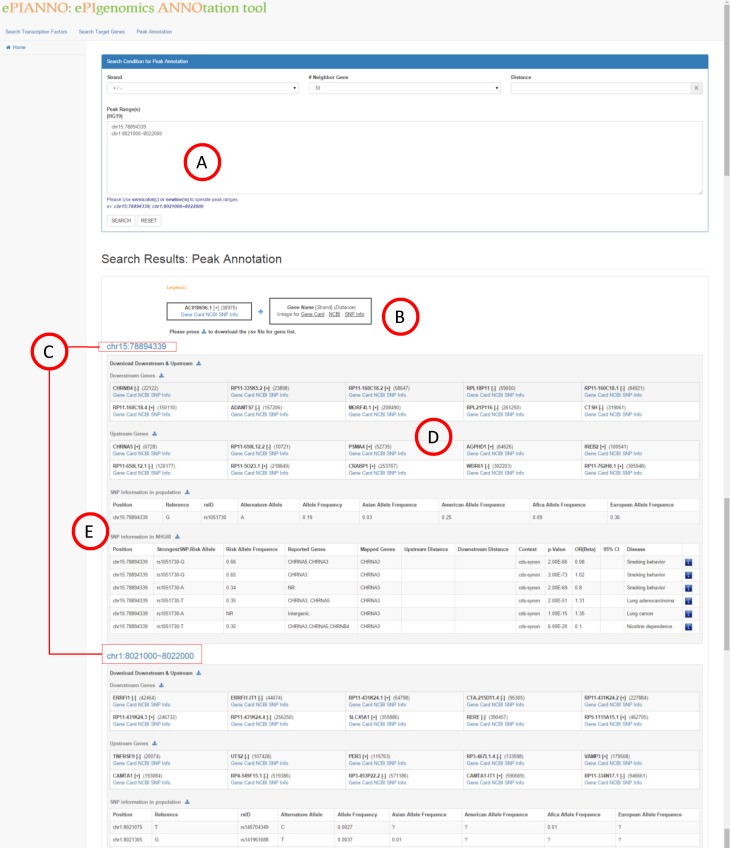
Peak annotations. We assume that users have aimed on some specific regions and this function is best to help them to explore local information of these regions. User could define region(s) of interest (A), number of neighbour gene, or distance to the given region in the query page. Gene information including gene name, strand, distance to region of query, and links of annotations will be shown (B). Users could expand links (C) and the detail gene information will be shown on the webpage or download them in EXCEL CSV format (D). SNP information in population and disease are also provided (E).

Users may have their own DNA-binding location (peak data from user’s ChIP-seq experiments) in mind and want to know the annotations of neighbour regions of the peak. To meet this need, the third function of *e*PIANNO is to search for neighbour genes and the annotation of specific genomic regions. This function helps a user to explore genes located near to given genomic coordinates of interest. If users have aimed on some specific regions, then this function can help them to explore local information in these regions. User could define region(s) of interest (Fig 4A), the number of neighbour genes, or distance to the given region in the query page. *e*PIANNO shows the genes and SNPs located around the region in query, both upstream and downstream. Gene information including gene name, +/- strand, distance to queried genomic region, and links of annotations will be shown on the browser (Fig 4B). Users could expand links (Fig 4C) and the detail gene information will be shown on the webpage or download them in the EXCEL CSV format (Fig 4D). Population SNP information and associations with disease are also provided (Fig 4E).

### Example1: rs1051730(chr15:78894339;C>T) and SMARCA4

In a protein-DNA binding event, both protein and DNA were involved. Alternations of protein and DNA may both affect the binding event. As a consequence, if a genomic variant was found to be associated with some diseases, alternations of the correspondent DNA-binding protein may also be linked to similar diseases. In the following case, we demonstrate that a DNA-binding protein which targeted a disease-associated genomic variant was also a potential disease-associated protein.

In 2008 and 2009, a genomic variant which caused synonymous substitution in *CHRNA3*, rs1051730, was reported to be associated with lung cancer and lung adenocarcinoma in two independent GWASs, respectively [25, 26]. We use “Search transcription factors” function of *e*PIANNO to explore any potential transcription factor may bind on it. First, we just put “chr15:78894339” in the query box and select “Transcription factor or DNA-binding protein” as the ChIP type to conduct the search (Fig 2A). We found the binding regions of three subunits of RNA polymerase complex and two transcription factors, SMARCA4 and YY1, exactly flank this position (Fig 2D). This may imply that these DNA-binding proteins were also potentially associated with lung cancer. *YY1* had been reported as a known lung cancer associated gene [27–30]. In 2014, *SMARCA4* was identified to be one of tumour suppressor gene in the non-small cell lung cancer because inactivation of *SMARCA4* could promote cancer aggressiveness by altering chromatin organization [31]. In *e*PIANNO, this genomic variant was annotated as the *SMARCA4* binding site by one experiment using cellline Helas3.

### Example 2: rs35675666(chr1: 8021973;G>T) and *NFKB*

In 2011, a genomic variant, rs35675666, was identified to be associated with inflammatory bowel disease (IBD), including Crohn's disease and ulcerative colitis [32]. We use “Search transcription factors” function of *e*PIANNO to explore any potential transcription factor may bind on it. First, we put “chr1:8021973” in the query box (Fig 2A), select “ENCODE_standford” as the data source (Fig 2B), and search “Transcription factor or DNA-binding protein” CHiP type (Fig 2C). We found the binding regions of 32 transcription factors exactly flank this position (Fig 2D). Among them, 3 transcription factors, NFKB, HSF1, and HNF4A had been reported to be linked to IBD [33–35].

Alternations of the TF binding region may cause the expression dysregulation of the downstream gene. If a genomic variant occurred in the TF binding region was found to be associated with some diseases, the expression dysregulation of the downstream gene may also be associated with such diseases. As a demonstration, we used the “peak Annotation” function in the *e*PIANNO and search “chr1:8021973” for 20 nearby genes both upstream and downstream (Fig 4A). We found three genes locate nearby rs35675666 in 30K upstream or downstream: *DDX11L1*, *WASH7P*, and *TNFRSF9* (Fig 4C and 4D). DDX11L1 is a lncRNA with unknown function and *WASH7P* is a pseudogene, we turned to the gene *TNFRSF9* (chr1: 7975931~ 8003225; minus strand) which located 17K upstream of rs35675666. In 2012, this gene was reported as one candidate gene associated with IBD [36]. In 2014, another independent study of human intestinal T cell, which combined results of transcriptome and genomic variants to infer IBD associated features, showed that rs35675666 and expression of both *TNFRSF9* and *HNF4A* were associated with IBD [37].

### Example 3: Batch query in *e*PIANNO

**Fig 5:**
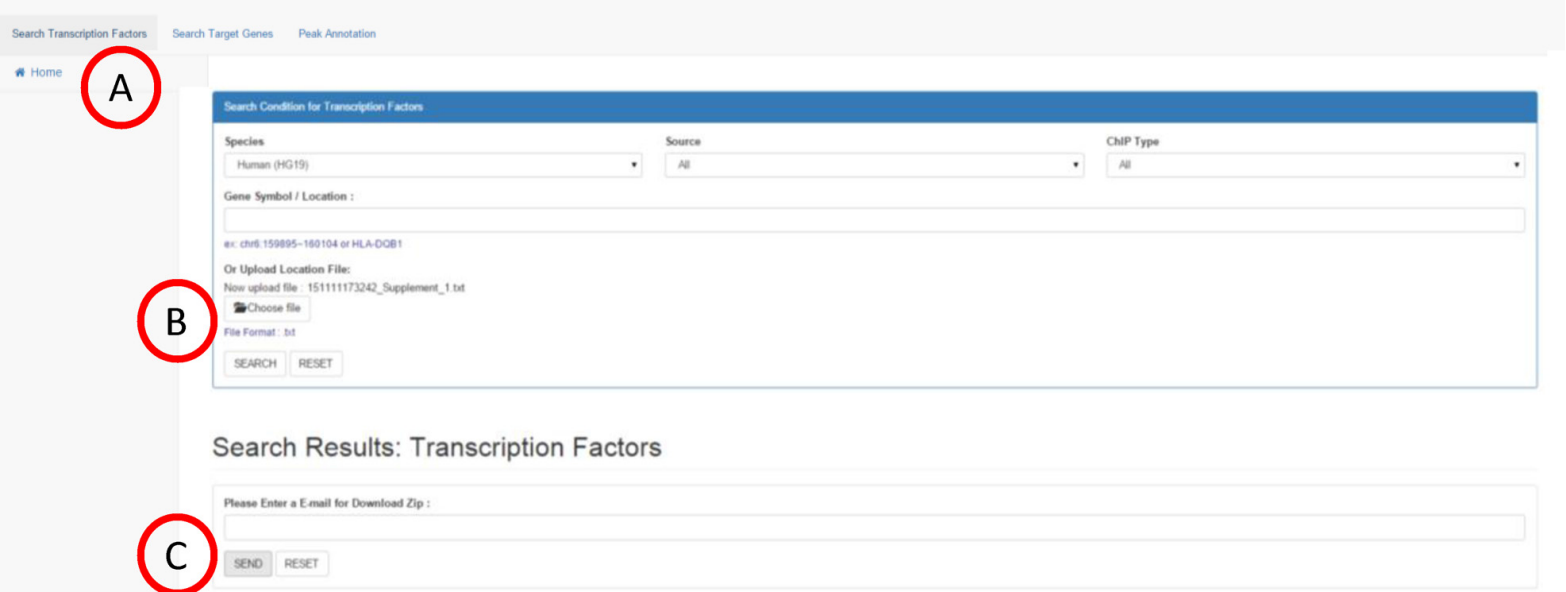
Batch query in ePIANNO. User could upload their queries in a text file instead of pasting on the website. For example, (A) user can choose “Search Transcription Factors”, (B) upload the sample peak file (S1 Text), (C) fill your email, and then click “SEND”. After *e*PIANNO finishing this job, one downloadable file will be shown on the page.

*e*PIANNO allowed users to upload their own peak data in TXT format or paste their peak regions in the query region and help them to add annotation. For example, (Fig 5A) user can choose “Search Transcription Factors”, (Fig 5B) upload the sample peak file by click “SEARCH” (S1 Text), (Fig 5C) fill your email and click “SEND”. After *e*PIANNO finishing this job, one compressed file will send email for user to download it. In this compressed file, each query region was saved in separated EXCEL files, respectively. In each EXCEL file, users could find the disease-associated SNPs, population SNP information, and peak data information. Currently, *e*PIANNO allowed maximum 300 query regions in each batch and each query region range within 100 kilo base pairs.

## Discussion

The main goal of *e*PIANNO is to help users to explore the associations between protein-binding event, disease-associated genomic variants, and information of general populations by annotating the ChIP-seq datasets. Currently, most ChIP-seq web services or depositories, such as CR Cistrome, ChIPBase, CistromeFinder, hmChIP, SwissRegulon, and CistromeMap, provided annotations and gene symbol query function. However, they did not provide batch query or region query functions. On the other hand, GWAS web services, such as GWAS Central and GWASdb, provided user-friendly interfaces for users to query disease-associated SNPs with batch query or region query functions. However, they did not provide the linkage between SNPs, peak information of protein-DNA binding, or information of general populations (1000 Genomes Project). As a consequence, *e*PIANNO was built to fill this gap.

Furthermore, *e*PIANNO allowed users to upload their own peak data in TXT format or paste their peak regions in the query region and help them to add annotation.

SNPs represent the most common type of genomic variation. SNPs in the coding region are most intensively studied since they are relatively easy interpretable with well-annotated protein-coding sequences of human genome. Some SNPs localized to the binding sites of various transcription factors may alter the regulation of gene transcription and exert functional effects. *e*PIANNO integrated the experiment results of ChIP-seq (hmChIP, ENCODE, and Roadmap Epigenomics), SNP information of populations (1000 Genomes Project), disease association information of GWAS (NHGRI), and gene annotation (ENCODE annotation). Users could not only search the relationship between TFs and target genes, but also explore the relevant genome variants and diseases. If users have their own genomic regions of interest, *e*PIANNO provides batch query methods, currently not provided by UCSC Genome Browser, (separated by semi-colon or another line) to explore genes and SNPs located in upstream or downstream with ENCODE gene annotations.

GWAS Catalog of NHGRI is the most comprehensive disease-SNP association database of the public domain. The 1000 Genomes Project contains most comprehensive genomic variants of populations worldwide. Genomic variants may contribute to binding affinity or other properties of protein-DNA interaction. In addition, some genomic variants are associated with known diseases. We demonstrated how users could use functions of *e*PIANNO webserver to explore useful information about TF related genomic variants. The example of SNP rs1051730 showed that DNA-binding proteins targeting a disease-associated genomic variant were also likely to be disease-associated genes. The example of SNP rs35675666 illustrated how *e*PIANNO helped establish the connection between the genomic variant of upstream TF-binding region, the associated disease and the TF expression. We believe that if users have their own disease-associated SNPs, may be from their own study, *e*PIANNO would be helpful to find out the potential relationship between SNPs and disease by annotating their roles at the epigenomic level.

Previous studies had used ChIP-seq data of ENCODE, GWASs Catalogs of NHGRI, and 1000 Genomes Project dataset or HAPMAP III dataset to investigate the relationships between SNPs, disease, and protein-DNA binding effects[18–20]. Results of these studies showed high quality statistical analysis and promising results. However, comprehensive information of ChIP-seq data (ENCODE, ROADMAP epigenomics, and hmChIP) and genomic variation (1000 Genomes Project) was still needed updated. In addition, previous studies showed that combinations of genomic variants and ChIP-seq data did provide valuable information for researchers. However, researchers without computational abilities cannot explore associations between their interesting TFs, binding sites, or genomic variations by themselves. Hence, a web based server with user friendly interface, easy input format, and output function would be useful for researchers without computational skills to obtain valuable information. As a consequence, we build this server *e*PIANNO with most update ChIP-seq datasets, the GWASs Catalog dataset, and annotations for users to query and annotate regions of their interest.

Genomic variants in the protein-DNA binding region might directly affect the binding strength of TFs or mode of chromatin modification at the transcription level. In addition, effects of the genomic variants might further reach the post-transcription level if the expressions of miRNA or lncRNA are affected. Although we did not give such complicate case of indirect effects of genomic variants in our examples, *e*PIANNO might help user to find out such case especially when they have their own protein-DNA binding regions of their own interest.

In *e*PIANNO, data of 1000 Genomes Project came from historical healthy subjects. However, data of NHGRI came mostly from various diseases and ChIP-seq data were generated from cells lines with various experimental conditions. As a consequence, combining data of annotations and ChIP-seq had its own limitations. Although *e*PIANNO helped users to explore potential associations between disease, protein-binding information, and their own regions of interest, these associations may not broadly applied in all cell types or in all experimental conditions. Results had to be evaluated by users’ aims and hypothesis of their studies. For an example, if lung cancer was considered in NHGRI database, cell line of ChIP-seq experiments was better to be lung cell lines. It may provide a more reasonable association between a SNP and a protein-binding site.

## Supporting Information

S1 Text Supporting text. (TXT)

## Abbreviations

NGS: next generation sequencing (NGS)
ChIP: the combination of chromatin immunoprecipitation
GWASs: genome-wide association studies
TFs: transcription factors
CRs: chromatin regulators
SNPs: single-nucleotide polymorphisms
IBD: inflammatory bowel disease
